# Crystal structure of (*E*)-3-(2-hy­droxy-4-methyl­phen­yl)-1-(2,4,6-tri­meth­oxy­phen­yl)prop-2-en-1-one

**DOI:** 10.1107/S2056989019011289

**Published:** 2019-08-30

**Authors:** Maadh Jumaah, Huey Chong Kwong, Melati Khairuddean

**Affiliations:** a School of Chemical Sciences, Universiti Sains Malaysia, Penang 11800 USM, Malaysia; b Department of Chemistry, Faculty of Science, Universiti Putra Malaysia, 43400 UPM Serdang, Selangor Darul Ehsan, Malaysia

**Keywords:** crystal structure, chalcone, enone bridge, O—H⋯O inter­action, data survey

## Abstract

The title chalcone derivative, C_19_H_20_O_5_, adopts a *trans* configuration with respect to the olefinic C=C double bond. The 2-hy­droxy-4-methyl­phenyl ring is coplanar with the attached enone bridge [torsion angle = −179.96 (14)°], where this plane is nearly perpendicular to the 2,4,6-tri­meth­oxy­phenyl ring [dihedral angle = 75.81 (8)°]. In the crystal, mol­ecules are linked into chains propagating along [010] by an O—H⋯O hydrogen bond. These chains are further connected into centrosymmetric dimer chains *via* weak C—H⋯O inter­actions. The conformations of related chalcone derivatives are surveyed and all of these structures adopt a skeleton with two almost orthogonal aromatic rings.

## Abstract 2

The title compound consists of a 2-hy­droxy-4-methyl­phenyl ring, a 2,4,6-tri­meth­oxy­phenyl ring and a prop-2-en-1-one connecting bridge. The overall conformations of the title compound are discussed and compared with those of related structures. In the crystal, mol­ecules are consolidated by O—H⋯O hydrogen bonds and weak C—H⋯O inter­actions.

## Chemical context   

Chalcones (1,3-di­aryl­prop-2-en-1-ones) are precursors of flavonoids and isoflavonoids in the plant kingdom (Ni *et al.*, 2004 ▸; Sahu *et al.*, 2012 ▸). Structurally, they consist of two aryl groups linked by an *α*, *β*-unsaturated ketone system (Ibrahim *et al.*, 2012 ▸; Kumar *et al.*, 2013 ▸), whereby the aryl groups can carry a variety of substituents such as hydroxyl, meth­oxy and alkenyl groups, which are by far the most commonly encountered ones in nature. With their structural simplicity and the associated ease of synthesis, chalcone compounds have attracted a considerable amount of attention because of their important pharmacological properties such as anti-oxidative (Aoki *et al.*, 2008 ▸), anti-inflammatory (Israf *et al.*, 2007 ▸), anti-gout (Jang *et al.*, 2014 ▸), anti-histaminic (Yamamoto *et al.*, 2004 ▸), anti-obesity (Birari *et al.*, 2011 ▸), anti-protozoal (Chen *et al.*, 1993 ▸), hypnotic (Cho *et al.*, 2011 ▸) and anti-spasmodic (Sato *et al.*, 2007 ▸) effects. In a continuation of our ongoing research on the properties of various chalcone derivatives (Sim *et al.*, 2017 ▸, Kwong *et al.*, 2018 ▸), we report herein the synthesis and crystal structure determination of the title compound, C_19_H_20_O_5_, (I).
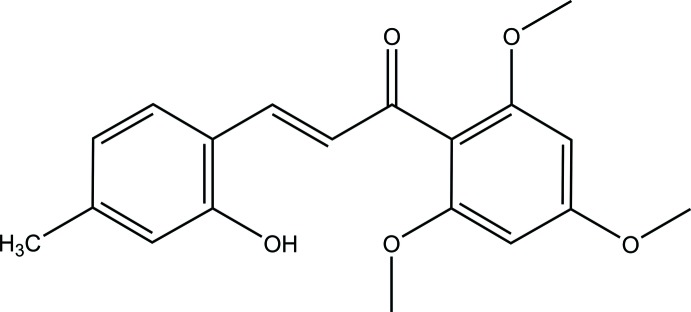


## Structural commentary   

**Figure 1:**
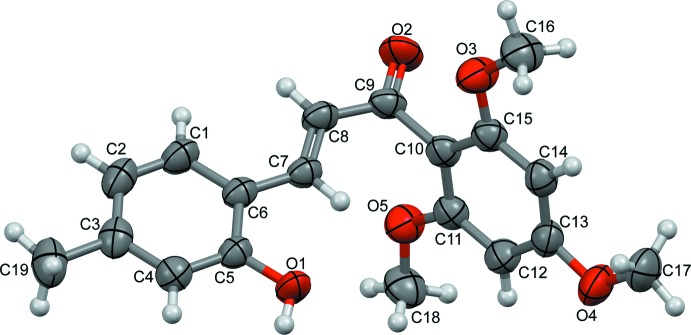
The title mol­ecule with the atom-labelling scheme and displacement ellipsoids drawn at the 50% ellipsoid probability level.

**Figure 2:**
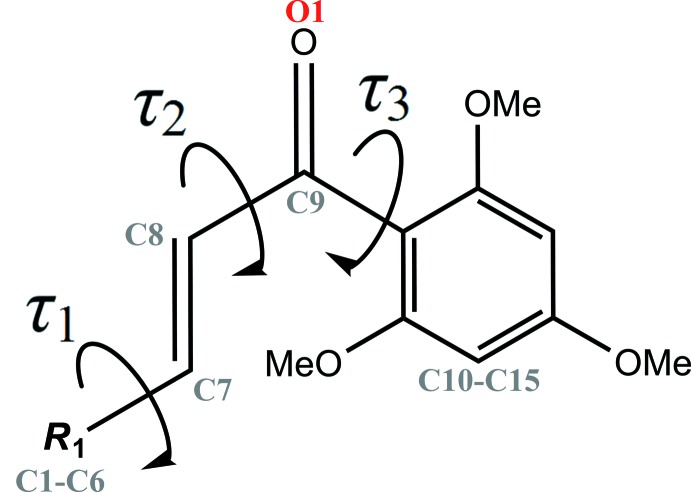
General chemical diagram showing torsion angles, *τ*_1_, *τ*_2_ and *τ*_3_.

The title chalcone derivative (I), crystallizes in the centrosymmetric triclinic space group *P* 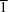 and its asymmetric unit consists of a single unique mol­ecule (Fig. 1 ▸). This mol­ecule is constructed of two substituted aromatic rings (2-hy­droxy-4-methyl­phenyl and 2,4,6-tri­meth­oxy­phen­yl) and an enone (–CH=CH—(C=O)–) connecting bridge. In the enone bridge, the C6—C7, C8—C9 and C9—C10 bond lengths of 1.446 (2), 1.441 (2) and 1.504 (2) Å, respectively, confirm their single-bond character, whereas the C7=C8 and C9=O2 bond lengths of 1.340 (2) and 1.2255 (17) Å, respectively, confirm the presence of a double bond. In addition, the C6—C7—C8 and C8—C9—C10 bond angles of 128.71 (13) and 119.47 (11)°, respectively, are consistent with the *sp* ^2^ hybridization of atoms C7, C8 and C9 (Kerr *et al.*, 2001 ▸; Loghmani-Khouzani *et al.*, 2009 ▸; Grealis *et al.*, 2013 ▸). As the olefinic double bond C7=C8 adopts a *trans* configuration [C6—C7—C8—C9 torsion angle = −179.96 (14)°], the structural conformation of (I) can be defined by three torsion angles. The torsion angles between the 2-hy­droxy-4-methyl­phenyl ring and the olefinic double bond (C5—C6—C7—C8, *τ*_1_), between the olefinic double bond and the carbonyl group (C7—C8—C9—C10, *τ*_2_) and between the carbonyl group and the 2,4,6-tri­meth­oxy­phenyl ring (C8—C9—C10—C11, *τ*_3_) are shown in Fig. 2 ▸. The torsion angles *τ*_1_ and *τ*_2_ are approximately ±180° or 0° [*τ*_1_ = −179.15 (14)° and *τ*_2_ = −0.8 (2)°], indicating that the 2-hy­droxy-4-methyl­phenyl ring and the enone bridge are coplanar. In contrast, the carbonyl group is nearly perpendicular to the attached 2,4,6-tri­meth­oxy­phenyl ring, as *τ*_3_ is 76.87 (19)°. In general, the mol­ecule of (I) can be considered as two individual planes, the first comprising the 2-hy­droxy-4-methyl­phenyl ring and the enone bridge [maximum deviation of 0.0021 (2) Å for atom C19], and the second the 2,4,6-tri­meth­oxy­phenyl ring [maximum deviation of 0.0059 (2) Å for atom C18]. These two mean planes form a dihedral angle of 75.84 (4)°, hence the mol­ecule of (I) possesses a skeleton with two almost orthogonal aromatic rings.

## Supra­molecular features   

**Figure 3:**
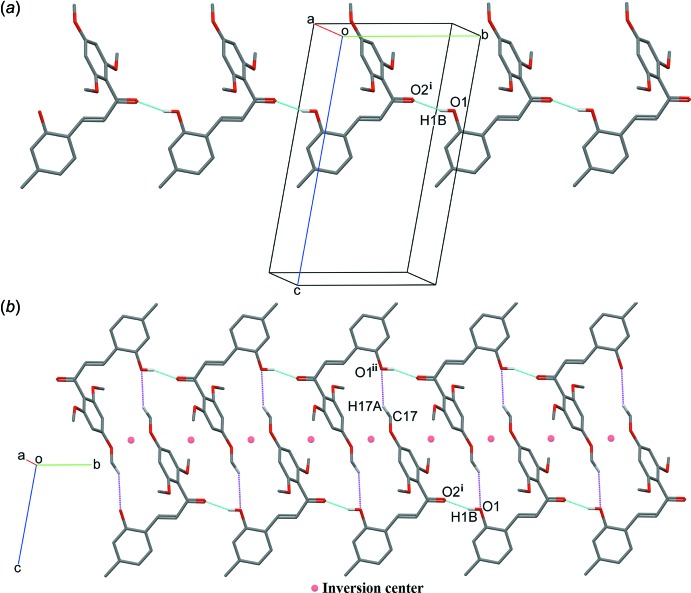
Views of (*a*) a chain of mol­ecules linked by O—H⋯O hydrogen bonds (shown as cyan dotted lines) and (*b*) a dimeric chain formed by weak C—H⋯O inter­actions (shown as magenta dotted lines). Symmetry code: (i) *x*, −1 + *y*, *z*; (ii) 1 − *x*, −*y*, −*z*. Hydrogen atoms not involved in these inter­actions are omitted for clarity.

In the crystal, the mol­ecules are linked into chains parallel to the *b* axis *via* classical O1—H1*B*⋯O2^i^ hydrogen bonds (Fig. 3 ▸ *a*). These chains are further connected into inversion-related dimeric chains by weak C17—H17*A*⋯O1^ii^ inter­molecular inter­actions (Fig. 3 ▸ *b*, Table 1 ▸).

## Database survey   

A search of the Cambridge Structural Database (CSD version 5.40, last update May 2019; Groom *et al.*, 2016 ▸) using (*E*)-3-phenyl-1-(2,4,6-tri­meth­oxy­phen­yl)prop-2-en-1-one as the reference moiety resulted in three chalcone structures containing 2,4,6-tri­meth­oxy­phenyl with different substituents. They include (*E*)-3-(***R*** **_1_**)-1-(2,4,6-tri­meth­oxy­phen­yl)prop-2-en-1-one, where ***R*** **_1_** = 2,4,6-tri­meth­oxy­phenyl (BAGXEN; Kerr *et al.*, 2001 ▸), 6-nitro­benzo[*d*][1,3]dioxol-5-yl (BUFMOF; Loghmani-Khouzani *et al.*, 2009 ▸) and 4-meth­oxy­phenyl (GESRAZ; Grealis *et al.*, 2013 ▸). As in (I), the mol­ecules of all these structures adopt a *trans* configuration with respect to C=C double bond (C6—C7—C8—C9 torsion angles = 175.5–179.1°). Although, *τ*_1_ for all of the structures indicates an *anti-periplanar* conformation (Table 2 ▸), in BUFMOF it deviates slightly from planarity (*τ*_1_ = 152.7°) whereas *τ*_1_ for the other mol­ecules is approximately 180° (*τ*_1_ = 174.1–176.0°, Table 2 ▸). Regarding the enone bridge, the torsion angle *τ*_2_ indicates that all of the structures are relatively planar (*τ*_2_ = −4.8–7.6°). The torsion angles *τ*_3_ always almost indicate a perpendicular arrangement (*τ*_3_ = 67.6–88.6°). This might arise from the steric repulsion between the carbonyl group and the attached 2,4,6-tri­meth­oxy­phenyl ring. This results in an overall L-shape for all of the structures, with the dihedral angle between the mean planes of the two aromatic rings being 61.6–80.4°.

## Synthesis and crystallization   

**Figure 4:**
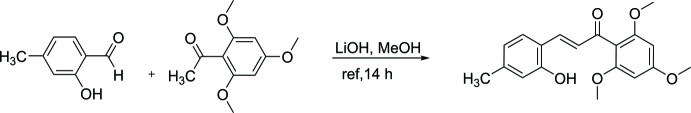
Reaction scheme for the synthesis of the title chalcone.

A reaction scheme for the synthesis of the title compound is given in Fig. 4 ▸. A solution of tri­meth­oxy­aceto­phenone (2 mmol) in 20 mL MeOH, LiOH (2.4 mmol) and 2-hy­droxy-4-methyl­benzaldehyde (1.6 mmol) was stirred at 368 K and the reaction progress was monitored by TLC. The reaction was quenched with diluted hydro­chloric acid to pH = 6 and extracted with ethyl acetate. The organic layer was washed with aqueous NaHCO_3_, water, and brine, successively. The organic layer was dried over anhydrous Na_2_SO_4_ and then concentrated to provide the product as a brown powder. The residue was purified by column chromatography with petroleum ether/ethyl acetate (10:1) as an eluent to afford the target compound (Yan *et al.*, 2016 ▸). Slow evaporation from an aceto­nitrile–water mixture provided X-ray quality crystals for the target chalcone compound.

**(** ***E*** **)-3-(2-hy­droxy-4-methyl­phen­yl)-1-(2,4,6-tri­meth­oxy­phen­yl)prop-2-en-1-one (I)**

Brown powder, yield 84.1%. m.p. 503–506 K. IR (cm^−1^): 3283 (O—H), 2929 and 2842 (C*sp* ^3^—H), 1603 (C=O), 1586 and 1457 (C=C). ^1^H NMR (500 MHz, DMSO-*d*_6_) δ, ppm: 10.02 (*s*, 1H), 7.45 (*d*, *J* = 16.2 Hz, 1H), 7.44 (*d*, *J* = 7.0 Hz, 1H), 6.86 (*d*, *J* = 16.2 Hz, 1H), 6.65 (*d*, *J* = 8.0 Hz, 2H), 6.30 (*s*, 2H), 3.83 (*s*, 3H), 3.70 (*s*, 6H), 2.23 (*s*, 3H). ^13^C-NMR (125 MHz, DMSO-*d*_6_) δ, ppm: 194.3, 162.2, 158.4, 157.1, 142.5, 140.2, 128.8, 127.9, 121.1, 118.9, 117.0, 111.9, 91.6, 56.2, 55.9, 21.6. CHN Elemental analysis: Calculated for C_19_H_20_O_5_: C, 69.50; H, 6.14; N. Found: C, 67.81; H, 5.72; N, 0.00.

## Refinement   

Crystal data, data collection and structure refinement details are summarized in Table 3 ▸ *.* C*-*bound H atoms were positioned geometrically (C—H *=* 0*.*93–0.96 Å) and refined using a riding model with *U*_iso_(H) = 1.2*U*_eq_(C) or 1.5*U*_eq_(C–meth­yl). The O-bound hydrogen was located from difference-Fourier maps and refined freely with O—H = 0.82 Å.

## Supplementary Material

Crystal structure: contains datablock(s) I. DOI: 10.1107/S2056989019011289/jj2214sup1.cif

Structure factors: contains datablock(s) I. DOI: 10.1107/S2056989019011289/jj2214Isup2.hkl

Supporting information file. DOI: 10.1107/S2056989019011289/jj2214Isup3.cml

CCDC reference: 1946810

Additional supporting information:  crystallographic information; 3D view; checkCIF report

## Figures and Tables

**Table 1:** Hydrogen-bond geometry (Å, °)

*D*—H⋯*A*	*D*—H	H⋯*A*	*D*⋯*A*	*D*—H⋯*A*
O1—H1*B*⋯O2^i^	0.82	1.88	2.6653 (15)	161
C17—H17*A*⋯O1^ii^	0.96	2.70	3.520 (2)	144

Symmetry codes: (i) 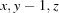; (ii) 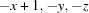.

**Table 2:** Selected torsion and dihedral angles (°) The dihedral angle is that between the mean planes of the aromatic rings.

Compound	***R*_1_**	*τ*_1_ (C5—C6—C7—C8)	*τ*_2_ (C7—C8—C9—C10)	*τ*_3_ (C8—C9—C10—C11)	Dihedral angle
(I)	2-hy­droxy-4-methyl­phen­yl	−179.2 (1)	−0.8 (2)	76.9 (2)	75.8 (1)
BAGXEN	2,4,6-tri­meth­oxy­phen­yl	174.1	−4.8	88.6	80.4
BUFMOF	6-nitro­benzo[*d*][1,3]dioxol-5-yl	153.7	6.8	67.6	61.6
GESRAZ	4-meth­oxy­phen­yl	176.0	7.6	−82.2	79.0

Note: values for the minor occupancy component of GESRAZ are not given.

**Table 3:** Experimental details

Crystal data	
Chemical formula	C_19_H_20_O_5_
*M*_r_	328.35
Crystal system, space group	Triclinic, *P* 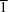
Temperature (K)	296
*a*, *b*, *c* (Å)	6.8072 (3), 8.5792 (4), 15.8010 (7)
α, β, γ (°)	100.365 (1), 99.433 (1), 104.984 (1)
*V* (Å^3^)	855.09 (7)
*Z*	2
Radiation type	Mo *K*α
μ (mm^−1^)	0.09
Crystal size (mm)	0.57 × 0.25 × 0.21

Data collection	
Diffractometer	Bruker APEXII DUO CCD area-detector
Absorption correction	Multi-scan (*SADABS*; Bruker, 2012 ▸)
*T*_min_, *T*_max_	0.908, 0.950
No. of measured, independent and observed [*I* > 2σ(*I*)] reflections	33448, 5018, 3199
*R*_int_	0.030
(sin θ/λ)_max_ (Å^−1^)	0.706

Refinement	
*R*[*F* ^2^ > 2σ(*F* ^2^)], *wR*(*F* ^2^), *S*	0.052, 0.171, 1.05
No. of reflections	5018
No. of parameters	217
H-atom treatment	H-atom parameters constrained
Δρ_max_, Δρ_min_ (e Å^−3^)	0.27, −0.19

Computer programs: *APEX2* and *SAINT* (Bruker, 2012 ▸), *SHELXS97* (Sheldrick, 2008 ▸), *SHELXL2013* (Sheldrick, 2015 ▸), *Mercury* (Macrae *et al.*, 2006 ▸) and *PLATON* (Spek, 2009 ▸).

## supplementary crystallographic information

### Crystal data

**Table:**

C_19_H_20_O_5_	*Z* = 2
*M**_r_* = 328.35	*F*(000) = 348
Triclinic, *P*1	*D*_x_ = 1.275 Mg m^−^^3^
*a* = 6.8072 (3) Å	Mo *K*α radiation, λ = 0.71073 Å
*b* = 8.5792 (4) Å	Cell parameters from 6606 reflections
*c* = 15.8010 (7) Å	θ = 2.5–25.7°
α = 100.365 (1)°	µ = 0.09 mm^−^^1^
β = 99.433 (1)°	*T* = 296 K
γ = 104.984 (1)°	Block, brown
*V* = 855.09 (7) Å^3^	0.57 × 0.25 × 0.21 mm

### Data collection

**Table:**

Bruker APEXII DUO CCD area-detector diffractometer	5018 independent reflections
Radiation source: fine-focus sealed tube	3199 reflections with *I* > 2σ(*I*)
Graphite monochromator	*R*_int_ = 0.030
φ and ω scans	θ_max_ = 30.1°, θ_min_ = 1.3°
Absorption correction: multi-scan (SADABS; Bruker, 2012)	*h* = −9→9
*T*_min_ = 0.908, *T*_max_ = 0.950	*k* = −12→12
33448 measured reflections	*l* = −22→22

### Refinement

**Table:**

Refinement on *F*^2^	0 restraints
Least-squares matrix: full	Hydrogen site location: inferred from neighbouring sites
*R*[*F*^2^ > 2σ(*F*^2^)] = 0.052	H-atom parameters constrained
*wR*(*F*^2^) = 0.171	*w* = 1/[σ^2^(*F*_o_^2^) + (0.0776*P*)^2^ + 0.1331*P*] where *P* = (*F*_o_^2^ + 2*F*_c_^2^)/3
*S* = 1.05	(Δ/σ)_max_ < 0.001
5018 reflections	Δρ_max_ = 0.27 e Å^−^^3^
217 parameters	Δρ_min_ = −0.19 e Å^−^^3^

### Special details

**Table:**

Geometry. All esds (except the esd in the dihedral angle between two l.s. planes) are estimated using the full covariance matrix. The cell esds are taken into account individually in the estimation of esds in distances, angles and torsion angles; correlations between esds in cell parameters are only used when they are defined by crystal symmetry. An approximate (isotropic) treatment of cell esds is used for estimating esds involving l.s. planes.

### Fractional atomic coordinates and isotropic or equivalent isotropic displacement parameters (Å^2^)

**Table:**

	*x*	*y*	*z*	*U*_iso_*/*U*_eq_	
O1	0.64130 (18)	0.04792 (12)	0.33273 (7)	0.0582 (3)	
H1B	0.622234	−0.051206	0.330607	0.087*	
O2	0.5594 (2)	0.71937 (13)	0.28620 (9)	0.0750 (4)	
O3	0.82818 (19)	0.58546 (15)	0.17162 (8)	0.0718 (4)	
O4	0.3336 (2)	0.11733 (16)	−0.04471 (7)	0.0741 (4)	
O5	0.18978 (18)	0.33623 (15)	0.22860 (8)	0.0653 (3)	
C1	0.7621 (3)	0.3898 (2)	0.52362 (11)	0.0587 (4)	
H1A	0.765072	0.500561	0.536913	0.070*	
C2	0.8168 (3)	0.3175 (2)	0.59089 (11)	0.0636 (4)	
H2A	0.856694	0.379893	0.648810	0.076*	
C3	0.8134 (2)	0.1518 (2)	0.57349 (10)	0.0576 (4)	
C4	0.7548 (2)	0.0621 (2)	0.48690 (10)	0.0536 (4)	
H4A	0.752792	−0.048531	0.474238	0.064*	
C5	0.6988 (2)	0.13422 (17)	0.41849 (9)	0.0462 (3)	
C6	0.7017 (2)	0.30075 (17)	0.43524 (9)	0.0461 (3)	
C7	0.6399 (2)	0.37021 (17)	0.36199 (9)	0.0462 (3)	
H7A	0.600214	0.297697	0.306638	0.055*	
C8	0.6322 (2)	0.52507 (17)	0.36314 (10)	0.0530 (4)	
H8A	0.670606	0.601882	0.417082	0.064*	
C9	0.5668 (2)	0.57758 (17)	0.28428 (10)	0.0506 (3)	
C10	0.5066 (2)	0.45590 (17)	0.19626 (10)	0.0474 (3)	
C11	0.3145 (2)	0.33383 (18)	0.16927 (10)	0.0496 (3)	
C12	0.2590 (2)	0.2222 (2)	0.08827 (10)	0.0553 (4)	
H12A	0.130137	0.141133	0.070877	0.066*	
C13	0.3996 (3)	0.23348 (19)	0.03331 (10)	0.0539 (4)	
C14	0.5911 (2)	0.35440 (19)	0.05781 (10)	0.0528 (4)	
H14A	0.683280	0.361673	0.020255	0.063*	
C15	0.6426 (2)	0.46422 (18)	0.13936 (10)	0.0502 (3)	
C16	0.9764 (3)	0.6078 (3)	0.11824 (14)	0.0747 (5)	
H16A	1.098130	0.697163	0.149806	0.112*	
H16B	1.014327	0.507669	0.103655	0.112*	
H16C	0.917128	0.633866	0.065061	0.112*	
C17	0.4721 (4)	0.1217 (3)	−0.10396 (12)	0.0798 (6)	
H17A	0.408006	0.034279	−0.156077	0.120*	
H17B	0.501338	0.226820	−0.119882	0.120*	
H17C	0.599756	0.106887	−0.075655	0.120*	
C18	−0.0021 (3)	0.2085 (3)	0.21029 (14)	0.0741 (5)	
H18A	−0.072792	0.225952	0.257352	0.111*	
H18B	−0.087708	0.210054	0.155938	0.111*	
H18C	0.024368	0.102923	0.205183	0.111*	
C19	0.8715 (3)	0.0699 (3)	0.64692 (12)	0.0785 (6)	
H19A	0.908022	0.148913	0.702583	0.118*	
H19B	0.755129	−0.021899	0.646508	0.118*	
H19C	0.988302	0.030616	0.638320	0.118*	

### Atomic displacement parameters (Å^2^)

**Table:**

	*U*^11^	*U*^22^	*U*^33^	*U*^12^	*U*^13^	*U*^23^
O1	0.0834 (8)	0.0397 (5)	0.0461 (6)	0.0191 (5)	0.0077 (5)	0.0007 (4)
O2	0.1156 (11)	0.0394 (6)	0.0798 (8)	0.0294 (6)	0.0388 (8)	0.0135 (5)
O3	0.0660 (7)	0.0665 (7)	0.0692 (8)	−0.0007 (6)	0.0305 (6)	−0.0029 (6)
O4	0.0803 (9)	0.0796 (8)	0.0461 (6)	0.0084 (7)	0.0123 (6)	−0.0022 (6)
O5	0.0577 (7)	0.0702 (7)	0.0659 (7)	0.0118 (5)	0.0271 (6)	0.0090 (6)
C1	0.0631 (9)	0.0534 (8)	0.0508 (8)	0.0140 (7)	0.0116 (7)	−0.0047 (7)
C2	0.0594 (9)	0.0766 (11)	0.0441 (8)	0.0141 (8)	0.0094 (7)	−0.0018 (7)
C3	0.0434 (8)	0.0790 (11)	0.0486 (8)	0.0153 (7)	0.0102 (6)	0.0146 (7)
C4	0.0527 (8)	0.0538 (8)	0.0543 (8)	0.0169 (7)	0.0119 (7)	0.0113 (7)
C5	0.0454 (7)	0.0463 (7)	0.0431 (7)	0.0118 (6)	0.0099 (6)	0.0030 (6)
C6	0.0432 (7)	0.0440 (7)	0.0464 (7)	0.0097 (5)	0.0117 (6)	0.0018 (6)
C7	0.0476 (7)	0.0406 (7)	0.0466 (7)	0.0112 (5)	0.0130 (6)	0.0009 (5)
C8	0.0607 (9)	0.0413 (7)	0.0523 (8)	0.0129 (6)	0.0163 (7)	−0.0009 (6)
C9	0.0586 (8)	0.0357 (6)	0.0600 (9)	0.0137 (6)	0.0246 (7)	0.0074 (6)
C10	0.0557 (8)	0.0403 (7)	0.0507 (8)	0.0185 (6)	0.0173 (6)	0.0108 (6)
C11	0.0525 (8)	0.0491 (8)	0.0530 (8)	0.0198 (6)	0.0169 (6)	0.0152 (6)
C12	0.0529 (8)	0.0563 (9)	0.0522 (8)	0.0117 (7)	0.0082 (7)	0.0113 (7)
C13	0.0638 (9)	0.0550 (8)	0.0426 (7)	0.0206 (7)	0.0073 (7)	0.0103 (6)
C14	0.0597 (9)	0.0568 (8)	0.0475 (8)	0.0214 (7)	0.0192 (7)	0.0136 (7)
C15	0.0539 (8)	0.0446 (7)	0.0543 (8)	0.0156 (6)	0.0169 (7)	0.0113 (6)
C16	0.0663 (11)	0.0762 (12)	0.0799 (13)	0.0099 (9)	0.0345 (10)	0.0137 (10)
C17	0.1025 (15)	0.0791 (12)	0.0497 (10)	0.0168 (11)	0.0256 (10)	0.0018 (8)
C18	0.0613 (10)	0.0826 (13)	0.0820 (13)	0.0132 (9)	0.0271 (9)	0.0297 (10)
C19	0.0684 (11)	0.1119 (16)	0.0570 (11)	0.0267 (11)	0.0087 (9)	0.0291 (10)

### Geometric parameters (Å, º)

**Table:**

O1—C5	1.3613 (16)	C8—H8A	0.9300
O1—H1B	0.8200	C9—C10	1.504 (2)
O2—C9	1.2255 (17)	C10—C15	1.390 (2)
O3—C15	1.3626 (19)	C10—C11	1.392 (2)
O3—C16	1.4151 (19)	C11—C12	1.383 (2)
O4—C13	1.3636 (18)	C12—C13	1.391 (2)
O4—C17	1.432 (2)	C12—H12A	0.9300
O5—C11	1.3651 (18)	C13—C14	1.385 (2)
O5—C18	1.419 (2)	C14—C15	1.384 (2)
C1—C2	1.371 (2)	C14—H14A	0.9300
C1—C6	1.403 (2)	C16—H16A	0.9600
C1—H1A	0.9300	C16—H16B	0.9600
C2—C3	1.392 (3)	C16—H16C	0.9600
C2—H2A	0.9300	C17—H17A	0.9600
C3—C4	1.381 (2)	C17—H17B	0.9600
C3—C19	1.511 (2)	C17—H17C	0.9600
C4—C5	1.387 (2)	C18—H18A	0.9600
C4—H4A	0.9300	C18—H18B	0.9600
C5—C6	1.3998 (19)	C18—H18C	0.9600
C6—C7	1.446 (2)	C19—H19A	0.9600
C7—C8	1.340 (2)	C19—H19B	0.9600
C7—H7A	0.9300	C19—H19C	0.9600
C8—C9	1.441 (2)		
			
C5—O1—H1B	109.5	C11—C12—C13	118.68 (15)
C15—O3—C16	119.21 (13)	C11—C12—H12A	120.7
C13—O4—C17	117.54 (14)	C13—C12—H12A	120.7
C11—O5—C18	118.63 (14)	O4—C13—C14	123.74 (14)
C2—C1—C6	121.65 (15)	O4—C13—C12	114.86 (14)
C2—C1—H1A	119.2	C14—C13—C12	121.39 (14)
C6—C1—H1A	119.2	C15—C14—C13	118.62 (14)
C1—C2—C3	120.75 (15)	C15—C14—H14A	120.7
C1—C2—H2A	119.6	C13—C14—H14A	120.7
C3—C2—H2A	119.6	O3—C15—C14	123.97 (13)
C4—C3—C2	118.51 (15)	O3—C15—C10	114.45 (13)
C4—C3—C19	120.07 (17)	C14—C15—C10	121.57 (14)
C2—C3—C19	121.41 (16)	O3—C16—H16A	109.5
C3—C4—C5	121.08 (15)	O3—C16—H16B	109.5
C3—C4—H4A	119.5	H16A—C16—H16B	109.5
C5—C4—H4A	119.5	O3—C16—H16C	109.5
O1—C5—C4	121.92 (13)	H16A—C16—H16C	109.5
O1—C5—C6	117.19 (12)	H16B—C16—H16C	109.5
C4—C5—C6	120.89 (13)	O4—C17—H17A	109.5
C5—C6—C1	117.12 (14)	O4—C17—H17B	109.5
C5—C6—C7	118.98 (12)	H17A—C17—H17B	109.5
C1—C6—C7	123.89 (13)	O4—C17—H17C	109.5
C8—C7—C6	128.71 (13)	H17A—C17—H17C	109.5
C8—C7—H7A	115.6	H17B—C17—H17C	109.5
C6—C7—H7A	115.6	O5—C18—H18A	109.5
C7—C8—C9	122.77 (13)	O5—C18—H18B	109.5
C7—C8—H8A	118.6	H18A—C18—H18B	109.5
C9—C8—H8A	118.6	O5—C18—H18C	109.5
O2—C9—C8	122.17 (14)	H18A—C18—H18C	109.5
O2—C9—C10	118.36 (14)	H18B—C18—H18C	109.5
C8—C9—C10	119.47 (12)	C3—C19—H19A	109.5
C15—C10—C11	118.35 (13)	C3—C19—H19B	109.5
C15—C10—C9	120.27 (13)	H19A—C19—H19B	109.5
C11—C10—C9	121.37 (13)	C3—C19—H19C	109.5
O5—C11—C12	124.18 (14)	H19A—C19—H19C	109.5
O5—C11—C10	114.43 (13)	H19B—C19—H19C	109.5
C12—C11—C10	121.39 (14)		
			
C6—C1—C2—C3	−0.3 (3)	C18—O5—C11—C12	5.6 (2)
C1—C2—C3—C4	0.4 (2)	C18—O5—C11—C10	−174.96 (14)
C1—C2—C3—C19	−179.49 (16)	C15—C10—C11—O5	−179.09 (12)
C2—C3—C4—C5	−0.5 (2)	C9—C10—C11—O5	0.4 (2)
C19—C3—C4—C5	179.37 (14)	C15—C10—C11—C12	0.4 (2)
C3—C4—C5—O1	179.78 (14)	C9—C10—C11—C12	179.85 (13)
C3—C4—C5—C6	0.5 (2)	O5—C11—C12—C13	179.48 (13)
O1—C5—C6—C1	−179.66 (13)	C10—C11—C12—C13	0.1 (2)
C4—C5—C6—C1	−0.3 (2)	C17—O4—C13—C14	0.2 (2)
O1—C5—C6—C7	1.36 (19)	C17—O4—C13—C12	−179.57 (15)
C4—C5—C6—C7	−179.32 (13)	C11—C12—C13—O4	179.14 (13)
C2—C1—C6—C5	0.2 (2)	C11—C12—C13—C14	−0.6 (2)
C2—C1—C6—C7	179.15 (14)	O4—C13—C14—C15	−178.99 (14)
C5—C6—C7—C8	−179.68 (14)	C12—C13—C14—C15	0.8 (2)
C1—C6—C7—C8	1.4 (2)	C16—O3—C15—C14	3.0 (2)
C6—C7—C8—C9	−179.96 (14)	C16—O3—C15—C10	−178.38 (15)
C7—C8—C9—O2	179.67 (15)	C13—C14—C15—O3	178.26 (14)
C7—C8—C9—C10	−0.8 (2)	C13—C14—C15—C10	−0.3 (2)
O2—C9—C10—C15	75.90 (19)	C11—C10—C15—O3	−178.96 (13)
C8—C9—C10—C15	−103.68 (16)	C9—C10—C15—O3	1.6 (2)
O2—C9—C10—C11	−103.56 (17)	C11—C10—C15—C14	−0.3 (2)
C8—C9—C10—C11	76.87 (19)	C9—C10—C15—C14	−179.73 (13)

### Hydrogen-bond geometry (Å, º)

**Table:**

*D*—H···*A*	*D*—H	H···*A*	*D*···*A*	*D*—H···*A*
O1—H1*B*···O2^i^	0.82	1.88	2.6653 (15)	161
C17—H17*A*···O1^ii^	0.96	2.70	3.520 (2)	144

Symmetry codes: (i) *x*, *y*−1, *z*; (ii) −*x*+1, −*y*, −*z*.
